# Integration of physical and genetic maps of common bean through BAC-derived microsatellite markers

**DOI:** 10.1186/1471-2164-11-436

**Published:** 2010-07-16

**Authors:** Juana M Córdoba, Carolina Chavarro, Jessica A Schlueter, Scott A Jackson, Matthew W Blair

**Affiliations:** 1 International Center for Tropical Agriculture (CIAT) Bean Project; A.A. 6713, Cali, Colombia; 2 Purdue University, Department of Agronomy, Indiana, United States: 915 West State Street West Lafayette, IN 47907-4778 USA; 3 University of North Carolina at Charlotte, Department of Bioinformatics and Genomics, Charlotte, United States: 9201 University City Blvd. Charlotte, NC 28223 USA

## Abstract

**Background:**

Common bean (*Phaseolus vulgaris *L.) is the most important legume for direct human consumption and the goal of this study was to integrate a recently constructed physical map for the species with a microsatellite based genetic map using a BAC library from the genotype G19833 and the recombinant inbred line population DOR364 × G19833.

**Results:**

We searched for simple sequence repeats (SSRs) in the 89,017 BAC-end sequences (BES) from the physical map and genetically mapped any polymorphic BES-SSRs onto the genetic map. Among the BES it was possible to identify 623 contig-linked SSRs, most of which were highly AT-rich. A subgroup of 230 di-nucleotide and tri-nucleotide based SSR primer pairs from these BACs was tested on the mapping parents with 176 single copy loci and 114 found to be polymorphic markers. Of these, 99 were successfully integrated into the genetic map. The 99 linkages between the genetic and physical maps corresponded to an equal number of contigs containing a total of 5,055 BAC clones.

**Conclusions:**

Class II microsatellites were more common in the BES than longer class I microsatellites. Both types of markers proved to be valuable for linking BAC clones to the genetic map and were successfully placed across all 11 linkage groups. The integration of common bean physical and genetic maps is an important part of comparative genome analysis and a prelude to positional cloning of agronomically important genes for this crop.

## Background

Common bean (*Phaseolus vulgaris *L.) is a member of the legume family (Leguminosae) and is one out of the five cultivated species from the *Phaseolus *genus that was domesticated by Native American populations [1]. In the case of common bean, domestication occurred in two different regions, namely Mesoamerica and the Andes of South America which resulted in the Andean and Mesoamerican gene pools [2]. Broad adaptation, consumer-acceptability and genetic diversity has made common bean the most widely grown legume for direct human consumption, with a worldwide distribution and presence in tropical, subtropical and temperate countries and many different environments. Furthermore, common bean is the third most commonly-grown grain legume behind only the protein and oil crops soybean (*Glycine max*) and peanut (*Arachis hypogea*). Common bean is highly nutritious with almost twice the protein levels of cereals, lower fats than soybean or peanut and higher amounts of lysine, phosphorus, iron, zinc, magnesium, copper and calcium than cereals [3]. In many developing countries, especially those of Latin America and Eastern and Southern Africa, common bean is a principal staple to the diets of the rural and urban poor [4].

Microsatellites, also known as simple sequence repeats (SSRs) are tandem repeats made up of motifs of up to six bp and are favored for the development of PCR based molecular markers due to their high rate of polymorphism [5,6]. Microsatellite loci can be classified according to their motif or to the number of repeats they contain: SSRs can be perfect having only one repeat type, or imperfect characterized by having repetitions interrupted by one or more nucleotides differing from the ones of the repetition. Other SSRs are compound with different combinations of perfect and imperfect repeats [6] or simple, when they are not compound. When considering the number of repeats, SSRs can also be classified into two types: class I with more than 10 repeats and class II with fewer than 10 repeats [7]. In common bean, microsatellites have been used for molecular characterization of cultivated and wild accessions as well as for genetic diversity analysis and anchoring of genetic maps (for example [8-11]).

Genetic maps based on molecular markers have been a standard of molecular biology studies in plants since the late 1980s and primarily since the start of the millennium these have been complemented by physical maps based on assembly of large-insert libraries. Physical maps were first constructed with yeast artificial chromosome clones but due to ease of DNA cloning and manipulation, bacterial artificial chromosome (BAC) clones are now preferred [12]. A physical map is a linear arrangement of DNA fragments that can be constructed using various methodologies. The most common are based on BAC fingerprinting and consist of complete digestion of the clones with one or more restriction enzyme, separation on agarose or polyacrylamide and bioinformatic assembly of overlapping clones through software programs such as FPC [13]. Shotgun clones are also used for physical map construction but are less robust than BAC-by-BAC characterization. Physical and genetic maps are not directly comparable because each uses a different distance measure. In the case of a physical map, distance is given in kilobases (kb) or megabases (Mb) while for the genetic map it is given in cM and is related to genetic recombination rates in a reference mapping population. Furthermore, the kb/cM ratio is not constant and varies between species' genomes [14] and each plant chromosome [15]. A physical map was constructed for common bean with a 12× coverage BAC library from CIAT, automated fingerprinting and FPC assembly at AGI/Purdue [16].

Different methods have been used to integrate the physical and genetic maps and can be grouped into the following: 1) *in silico *comparison of marker sequences to a whole genome sequence, as done for rice [17]; 2) BAC pooling and PCR screening as done for sorghum (*Sorghum bicolor *L.) [18] and soybean (*Glycine max *L. Merr.) [7]; 3) hybridization using overgo probes as done for various crops [19-21] and 4) mapping of molecular markers from BAC-end sequences as performed in soybean [22,7], *Medicago truncatula *[23], rice [24,25] and grape [26]. In this last method, the most common and useful molecular markers have been microsatellites, which can be found in BAC-end sequences (BES) and used for genetic mapping to link BACs from a physical map. The objectives in this study were 1) to develop BES-SSR markers from the BAC-end sequences for the G19833 BAC library, 2) to use these as microsatellite markers or sequence tagged connectors (STCs) on the genetic map of the recombinant inbred line population DOR364 × G19833 where one parent matched the library source, and 3) to produce an integrated genetic/physical map for the species.

## Results

### Identification of SSR motifs in the BAC-ends

A total of 875 microsatellites were identified in the 89,017 BAC-end sequences from the physical mapping project presented in Schlueter et al. [16]. These microsatellites were named using a combination of the series name BMb with a sequential number, and organized according to the contig or singletons they were derived from, their repetition type and the motif they contained (See Additional File 1: Information about the BMb microsatellite loci used for primer pair development). Among the microsatellites identified in the BES evaluation, 623 were associated with BAC contigs and were considered further while 252 were from singletons and were not used. All the microsatellites were perfect, meaning they had just one motif.

In the 623 contigged SSRs identified, di-nucleotide (44%) and tri-nucleotide (28%) motifs were more common than tetra-nucleotide (13%) and penta-nucleotide (15%) motifs. The most common di-nucleotide motif was AT/TA (75%), followed by AG/TC (20%) while AC/TG microsatellites were uncommon. Among the tri-nucleotides, the most frequent motifs were A/T rich, especially ATA/TAT (46%) and AGA/TCT (29%). In contrast, the ACT/TGA, AGC/TCG and CGC/GCG motifs were much less frequent and all together accounted for only 6% of the SSRs found. Among all the motif types, most of the SSRs (58%) belonged to Class II (less than 10 repeats) with the remainder of SSRs were from class I, these being mainly of the motifs AT/TA (70.5%), AG/TC (17%) and ATA/TAT (8%).

### BES-SSR polymorphism screening

Prior to the screening of the parental genotypes DOR364 and G19833, a group of 230 microsatellites were selected from the BAC-end SSRs identified above so as to focus on the SSR motifs that were most likely to show polymorphism and to be useful in linking the greatest number of BAC clones to the genetic map. Selection was based on repeat type with priority given to di-nucleotides and tri-nucleotides that were A/T rich and had five or more repetitions; and on the SSRs being located in BAC contigs rather than in singletons. In addition, an effort was made to select only one SSR locus per contig from the physical map. As a result, the subgroup of 230 selected SSRs covered an equal number of contigs with 75% of the BES-SSRs selected corresponding to AT/TA and ATA/TAT motifs, 19% to AG/TC or AGA/TCT motifs and the rest having the motifs AC/TG, TCA/AGT, GGT/CCA and AGC/TCG. In terms of microsatellite class, 168 BES-SSRs belonged to class I and 62 to class II.

In the molecular characterization of the new SSRs in the parental survey, all the SSRs were single copy and the amplified products had the expected size, the amplification success rate was 76.5% (176/230) and the calculated polymorphism rate was 65% (114/176). The 54 SSR primer pairs that presented amplification problems such as lack of amplification, multiple banding, stuttering or unexpected size amplification products were not used for further analysis. No relation was found between these amplification difficulties and the repeat motifs. Furthermore the 176 successful primer pairs represented all the SSR motifs described above. When microsatellites of different motif sizes were compared for their polymorphism rate (Table 1), the di-nucleotide based loci were somewhat more polymorphic at 70% than tri-nucleotides based loci at 47%. Similarly, class I microsatellites were more polymorphic on average (70%) with 88 out of 125 SSRs showing allelic difference compared to class II microsatellites (63%) with only 26 out of 41 with allele differences. In terms of the individual motifs, AT/TA motif microsatellites were highly polymorphic (77%).

**Table 1:** Characteristics of the BES-SSR identified

Repeat type	**Motif**^**1**^	Total SSR	**Monomorfic**^**2**^		Polymorfic	
			**Class I**^**3**^	Class II	Class I	Class II
Di-nucleotide	**AT/TA**	100	20% (20)	3% (3)	67% (67)	10% (10)
	**AC/TG**	5	40% (2)	20% (1)	40% (2)	-
	**AG/TC**	27	40.7% (11)	7.4% (2)	33.3% (9)	18.5% (5)
**Subtotal**		**132**	**25% (33)**	**4.5% (6)**	**59.1% (78)**	**11.4% (15)**

Tri-nucleotide	**ATA/TAT**	24	16.6% (4)	37.5% (9)	29.2% (7)	16.7% (4)
	**AGA/TCT**	14	-	35.7% (5)	21.4% (3)	42.8% (6)
	**AGC/TCG**	2	-	100% (2)	-	-
	**AGT/TCA**	3	-	66.7% (2)	-	33.3% (1)
	**GGT/CCA**	1	-	100% (1)	-	-
**Subtotal**		**44**	**9.1% (4)**	**43.2% (19)**	**22.7% (10)**	**25% (11)**

**TOTAL**		**176**	**21% (37)**	**14.2% (25)**	**50% (88)**	**14.8% (26)**

^1 ^Di- and tri-nucleotide motifs considered for the polymorphism survey

^2 ^Percentages and numbers of microsatellites in each SSR class and for each motif which were monomorphic or polymorphic in the survey of the DOR364 × G19833 population parents

^3 ^Class I: motifs longer than 10 repeats. Class II: motifs shorter than 10 repeats.

### Genetic mapping of BES-SSRs

**Figure 1:**
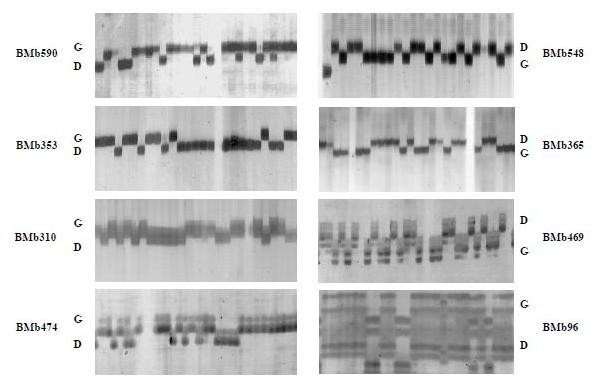
**Representative BMb markers**. BES-SSR markers scored in a subset of recombinant inbred lines of the mapping population based on the cross DOR364 (D) × G19833 (G). Markers BMb590, BMb548, BMb353 and BMb365 have AT motifs, BMb310 has an ATA motif and BMb469, BMb474 and BMb96 represent the motifs AGA, CT and CA, respectively.

The 114 polymorphic BMb markers identified in the parental screening were scored in the recombinant inbred line mapping population based on the cross DOR364 × G19833 (Figure 1) and integrated into the genetic map for this population from Blair et al. [8]. Integration was successful with a total of 99 new BMb markers mapped into the genetic map with a high LOD score. Molecular markers mapping with a LOD below 3.0 or unassigned to established linkage group were excluded from the map. For example, the SSR markers BMb35, BMb162, BMb214, BMb283, BMb363, BMb365 and BMb535 could not be mapped since they were assigned to more than one linkage group with equivalent LOD scores. The markers BMb483, BMb424, BMb545 were assigned to only one linkage group but their LOD scores were lower than 3.0. Finally another set of markers (BMb25, BMb192, BMb246, BMb275 and BMb422) presented distances from neighboring markers longer than 20 cM and therefore were not included given the high saturation of the map.

The new genetic map which included 116 previously mapped SSR loci from Blair et al. [8,10] was found to cover 1,397 cM and had a total of 215 SSRs all together with an average distance between neighboring loci of 6.6 cM (Table 2). Linkage group lengths ranged from 171.8 cM (b09k) to 80.8 cM (b07a) and a greater number of BMb loci were placed on b08f (14), and b02d (12) and a lower number on b06g (5) compared to other linkage groups. Despite this, the distribution of the BMb loci was found to be random across all linkage groups according to a chi-square test (χ^2 ^= 0.87 p > 0.05). While most linkage groups had close to the average of 9 BMb loci per linkage group, BMb loci were predominant on linkage groups b01h, b08f and b10j compared to previous SSR loci. Finally, segregation distortion was observed for 27 out of the 99 newly-mapped loci, this means that the expected ratio of 1:1 was not observed in the progeny for these markers with chi-square tests at p = 0.05. Most of these loci with segregation distortion were mapped on linkage groups b08f, b03c or b02d. Segregation distortion was towards the parental genotype G19833 on linkage group b01h, b02d, b06g and b08f, while in b03c, b07a and b09k segregation distortion was towards DOR364.

**Table 2:** General information about the integrated common bean genetic/physical map including number of BMb or other SSR markers placed in each linkage group, length of the linkage group and number of megabases (Mb) anchored to each of these

Linkage Group	BMb markers	Other SSR markers	Total SSR markers	Genetic Length (cM)	Anchored contigs (Mb)
**b01h**	10	8	18	145.8	4.8
**b02d**	12	22	34	168.3	6.8
**b03c**	10	10	20	142.2	3.9
**b04b**	7	13	20	133.1	3.3
**b05e**	7	8	15	136.2	4.3
**b06g**	5	7	12	85.77	1.3
**b07a**	9	9	18	80.8	4.4
**b08f**	14	7	21	111.8	3.8
**b09k**	8	11	19	171.8	1.7
**b10j**	10	7	17	112.8	8.0
**b11i**	7	14	21	103.9	4.8

**TOTAL**	99	116	215	1397	47.1

### Integrated genetic/physical map for common bean

**Figure 2:**
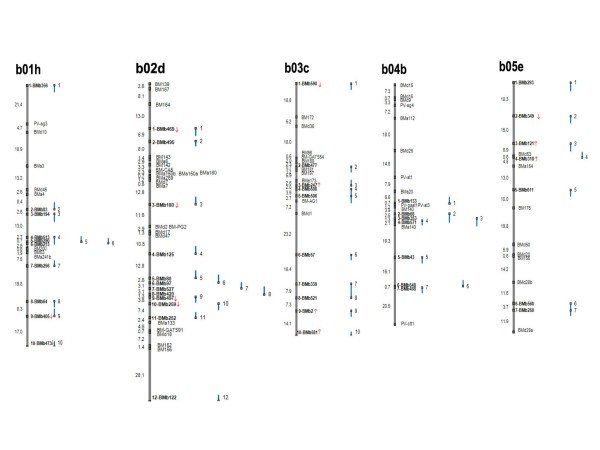
**Integrated common bean genetic/physical map; linkage groups b01 to b05**. Integration of the physical and genetic maps using BES-SSRs (shown in bold). Segregation distortion is represented by red arrows, upwards means distortion to DOR364 and downwards is to G19833.

**Figure 3:**
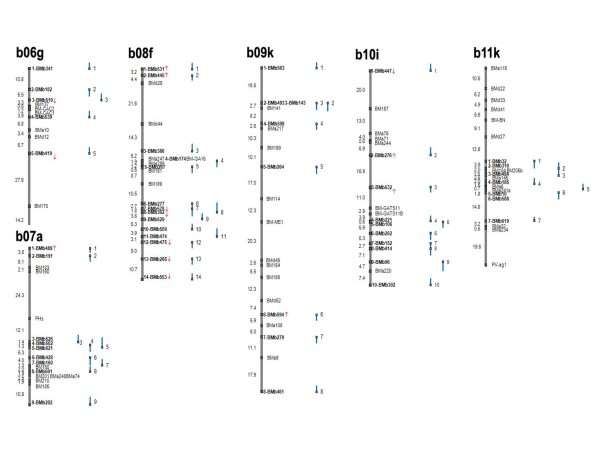
**Integrated common bean genetic/physical map; linkage groups b06 to b11**. Integration of the physical and genetic maps using BES-SSRs (shown in bold). Segregation distortion is represented by red arrows, upwards means distortion to DOR364 and downwards is to G19833.

The information from genetic mapping of the BES-SSRs was then used to create an integrated genetic and physical map for the common bean genome. This integrated map is presented in Figures 2 and 3 with various components shown diagrammatically: for example to the far left of the figure is the genetic map shown as a continuous line with genetic distances in cM. The physical map is shown as a series of smaller lines adjacent to the genetic map, representing anchored BAC clones and their corresponding contigs. Anchoring points between both maps are depicted as grey squares representing mapped SSR loci on the genetic map and BAC-ends. Integration points are numbered sequentially from the top to the bottom of the linkage group and orientation of the linkage groups follows Blair et al. [8]. The BAC clones and corresponding contigs associated with each anchoring microsatellite marker are given for the 99 linkage points throughout the genome (See Additional File 2: Information about the components of the integrated common bean map).

To facilitate interpretation of the physical linkages with the genetic map, we represented each BAC clone as a line that is proportional to its length and showed whether the BES-SSR was anchored to the forward/5' or reverse/3' BAC-end by orientation of the grey box at the top or bottom of the line, respectively. Finally, for consistency between new BMb markers and the BM, BMc and BMd markers mapped by Blair et al. [10], the ATA rich markers from Blair et al. [8] are re-named as BMa markers with the same numerical identity as in that previous study. Additionally, some of these BMa markers were remapped to their ideal locations based on the higher saturation of this genetic map. In terms of the physical coverage of the integrated genetic map, a total of 5,055 individual BAC clones were represented by the 99 contigs linked to the genetic map. The average number of contigged BACs per BES-SSR anchor point was 51.

## Discussion

### SSR motif prevalence and repeat length in common bean

A large number of SSRs were identified in the BAC-end sequences in this study with the most common microsatellites having di-nucleotide repeats rather than tri-, tetra- or penta-nucleotide repeats. The most common motifs were those rich on adenine and thymine especially AT/TA and ATA/TAT. The prevalence of di-nucleotide over tri-nucleotide or other SSRs was observed previously in the common bean genome by various authors [27-31] and also may be characteristic of cowpea (*Vigna unguiculata*) [31], soybean [32] and chickpea (*Cicer arietinum *L.) [33]. Similarly, the high frequency of A/T rich motifs among SSRs in common bean agrees with results of Métais et al. [34] and Blair et al. [8] and appears to be characteristic of legumes. In other plants, the microsatellites (AT)n, (AG)n, (AAG)n and (A)n are prevalent [6,35].

In terms of the predominant microsatellite class, most di-nucleotides were class I (longer than 10 repetitions) as seen in other legumes species such as *Medicago*, *Lotus *and soybean [36] but most tri-nucleotide based loci were class II. Repeat length may be constrained by evolutionary forces as has been seen in the human genome where deleterious mutations can occur as a consequence of the expansion or contraction of very long SSR repeats [37]. The maximum repeat length for the BES-SSRs in this study even among class I AT/TA di-nucleotide loci was 72 bp suggesting that ultra-long SSRs are uncommon in BAC-end sequences from *P. vulgaris*. This is in agreement with previously results for genomic microsatellites obtained by Hanai et al. [30] and Blair et al. [8] with AT rich microsatellites with the exception of BMa20 which was longer than 80 nt. The rate of mutation in (AT)n microsatellites was found to be higher than for (AG)n microsatellites in *Drosophila melanogaster *due to a bias in the mismatch repair mechanism against AT motifs which led to more variable and longer SSRs for this motif [38]. A similar situation could take place in plants, although more specific studies are necessary.

### SSR polymorphism was related to motif type rather than to motif length

Overall polymorphism rate in this study was related in the first place to SSR motif and secondly to SSR length. Higher polymorphism was seen in di-nucleotides in comparison with tri-nucleotides, while there was some tendency for long class I SSRs to be more polymorphic although this was not as pronounced as expected. This suggests that longer microsatellite are not necessarily preferential targets for replication slippage, unequal crossing-over or higher mutation rates compared to shorter microsatellites as has been suggested by previous studies [5,38,39]. It is also possible that tri-nucleotide or shorter motifs were preferentially found in gene-coding regions that would be more conserved but this was not analyzed for the BES since these are generally non-coding according to Schlueter et al. [16].

In an attempt to select highly polymorphic markers, we concentrated on A/T rich motif loci based on our success with this type of microsatellite in previous genetic mapping [8]. In general, AT/TA and ATA/TAT microsatellites were highly prevalent and highly polymorphic in the inter-genepool population, however, some amplification problems of these microsatellites may have been attributable to their palindromic sequences, low alignment temperature or high probability of self complementarity. Further studies could use AG/TC or AC/GA based microsatellites, but these have lower polymorphism rates in common bean than the AT rich microsatellites [8,9].

### Uniform distribution of BES-SSR loci across the common bean genome

In the genetic mapping phase of this project, we found the BES-SSR loci to be distributed across all linkage groups with no significant bias towards any specific chromosome although a slightly larger number of BES-SSR loci were found on linkage groups b02d and b08f. The good coverage of the BMb markers is in contrast to other SSR mapping studies in common beans especially for the (GA)n-based BM markers, the gene-based BMd markers [10] the AT-rich BMa markers [8] and to some extent other PV markers [40], all of which have tended to be more clustered. One of the goals of including new microsatellites in a previously constructed genetic map was to fill in map coverage especially in gaps from this previous mapping [8,10]. As a result, we wanted molecular markers that did not map together but rather mapped uniformly across the genome. The complementarities of the BES-SSR markers with previous mapping allowed us to supplement coverage on nearly all the linkage groups, but especially on b01h, b03c, b05e, b06g, b08f and b10j. As a result, the average number of microsatellites per linkage group in the DOR364 × G19833 map stands at 19.5 with all linkage groups having more than 15 SSR loci except for B06g. For some unknown reason linkage group b02d was heavily populated by microsatellites in this study as well as previously [8,10].

The uniformity of the BES-SSR loci across the genome may be related to the fact that the BAC-ends were a representative sample of the entire genome and to the similar and uniform physical size of common bean chromosomes, based on cytogenetic study by Pedrosa-Harand [41]. In that study, the authors assigned linkage groups to common bean chromosomes based on previously mapped single copy RFLP sequences used as FISH probes. The authors determined chromosome size based on FISH signal strength assuming a genome size of 637 Mb, and found that b01h, b03c, b07a and b08f all had similar sizes between 64 and 67 Mb, while b06g (44 Mb) had a smaller size and the rest were intermediate with sizes between 52 and 59 Mb. Tight correlation was not found between the physical length for each chromosome and the number of BES-SSR markers mapped for each linkage group in our study or the genetic length of the linkage group, meaning that the largest chromosomes did not have more SSRs or longer length as a genetic map. However, we have observed in the past that microsatellites from enriched libraries that target only a few motifs such as long GA/CT, CA/GT or ATA/TAT repeats [8,10,42] were biased towards certain chromosomes such as b02d and b04b [8,10] and towards specific chromosomal regions [8].

The uniform distribution of the BES-SSR markers would be the result of different factors. For example, the fact that we targeted various SSR motifs may have helped increase the chances of having randomly distributed markers [23]. In addition, the restriction enzyme used to generate the BAC library in the physical mapping project may have helped ensure an even genomic distribution. In our case, the BAC clones and resulting BES were from a *Hind*III derived BAC library for the Andean genotype G19833 [16]. Notably, *Hind*III is a type II site-specific enzyme with a fairly common restriction site therefore it is likely that distribution of the enzyme digestion sites may have contributed to eliminating biases in the BAC contigs generated. Likewise, the BES we sampled were from the entire library and therefore the eventual map location of BES-SSRs was random. These results also suggest, as previously postulated by Pedrosa-Harand et al. [43], that repeats can be interspersed with single copy sequences in regions different from pericentromeric heterochromatin.

Despite the random distribution, segregation distortion toward a specific genotype and assignment of distorted markers in a few clusters was observed. Segregation distortion is quite often observed in common bean as described by Blair et al. [10], Checa and Blair [44] and Frei et al. [45] and suggests the existence of incompatibility genes, or genes for sporophytic selection and gamete elimination in the species. In this study segregation distortion was observed for the linkage groups b02d, b03c and b08f, principally. For linkage group b02d the distorted loci were in the middle of the linkage group, while for b03c and b08f they were located distally.

### Integration of the genetic and physical maps

In the integration phase of this study, we used a method based on the use of BES-SSR anchoring points which was expected to be highly precise and accurate if the contig assembly had been performed well [22]. The BES-SSR markers used as anchor points allowed us to integrate 5,055 BAC clones through 99 contigs which together had a physical length of 47.1 Mb based on the FPC assembly, thus corresponding to 7.4% of the bean genome based on a genome size of 637 Mb [41].

The genome coverage of the integrated map of common bean could be increased by using a larger number of contig-based BES-SSR markers as was done for the integrated map of *Medicago truncatula *[23]. For example, if the 623 BES-SSR microsatellite loci had been screened, they potentially would have linked 20,861 BAC clones, almost half the library, while the 230 selected BES-SSRs represented contigs with 11,913 BAC clones from the fingerprinting described in Schlueter et al. [16]. The integrated common bean genetic/physical map is saturated enough to map QTL or genes to physical regions of the genome.

The integrated map, apart from being a resource for genetic mapping or positional cloning, could be used to find new linkages between contigs. In this case, only two BMb markers (BMb493 and BMb143) were close enough (0 cM) to postulate the possible overlap or close proximity of their constituent BAC clones. For the other BES-SSR markers, clear genetic separation showed that none of the assembled contigs overlapped with each other. Despite this, these results do not preclude the possibility of merging contigs through further genetic mapping.

## Conclusions

Apart from our objective of saturating the DOR364 × G19833 genetic map with BES-SSR markers, our other main goal was the integration of the physical map of common bean with this genetic map. The importance of an integrated physical and genetic map is in its ability to physically locate loci that are known to be polymorphic between mapping parents with a high degree of precision and accuracy to a set of contigged large-insert clones or sequences.

*In s****i****lico *method used for SSR identification in BAC-end sequences was a good option for obtaining widespread and evenly distributed markers of adequate polymorphism. The genetic map was saturated in SSRs and was easily linked to the physical map. It is pertinent to take into account the robustness of the integrated map obtained because the same genotype used for the physical map construction was one of the parents of the mapping populations used to place BES-SSR markers.

## Methods

### Identification of SSRs in the BAC-ends

A total of 89,017 BAC-end sequences produced as part of the physical mapping project described in Schlueter et al. [16] and originally from a BAC library of the Andean common bean genotype G19833 were searched for SSR repeats with BatchPrimer3 from You et al. [46]. This software has a flexible interface where the user can specify various parameters. In this case, the criterion used for the microsatellite search was a minimum of five repetitions for di-nucleotide motifs, four repetitions for tri-nucleotide motifs and three repetitions for tetra- or penta-nucleotide motifs. Primer design conditions were for a length of 18-23 nt and a melting temperature (T_m_) of 50-60°C. Primers were designed around the SSR motif such that the PCR product size would be between 100 and 300 base-pairs (bp).

### BES-SSR amplification

PCR reactions were carried out in a final volume reaction of 15 μl containing 20 ng of total genomic DNA, 0.15 μM each of the forward and reverse primers, 2.0 mM of MgCl_2_, 200 μM of total dNTP and 1 unit of *Taq *polymerase. The PCR program involved a touchdown profile with a hot start of 93°C for 3 min; followed by denaturation for 30 sec at 92°C; then annealing for 30 sec at the T_m _(+ 4°C) of the lower temperature primer and then extension for 45 sec at 72°C. A touchdown profile was used with a 1°C drop in extension temperatures per cycle for 8 cycles followed by 27 cycles of denaturation for 30 sec at 92°C, annealing for 30 sec at the T_m _(- 4°C) and extension for 45 sec at 72°C to ensure strong PCR products. Afterwards, there was a 5 min extension period at 72°C.

### SSR locus detection

After amplification, 5 μl of formamide containing 0.4% w/v bromophenol blue and 0.25% w/v xylene cyanol FF was added to each PCR reaction and the mixture was denatured at 96°C for 6 min. Subsequently, the mixtures were loaded with a multi-pipette into positions of a shark tooth comb set into 4% denaturing polyacrylamide (29:1 acrylamide:bis-acrylamide) gels that contained 5 M urea and 0.5× TBE. The gels were run in Owl Sequencing Units (Thermo Fisher Scientific Inc, Waltham MA) at a constant 50°C/100 W for approximately 1 hr. Detection of PCR amplification products was via silver staining according to Blair et al. [8-10] and the allele sizes were estimated based on 10, 25 and 50 bp MW ladders.

### Genetic and physical mapping

After the parental genotypes were scored for their alleles, any polymorphic microsatellites were mapped using the recombinant inbred line (RIL) population from Blair et al. [10] based on the cross DOR364 × G19833 where DOR364 is a Mesoamerican advanced breeding line from CIAT and G19833 is an Andean germplasm accession from Peru. This population is from an inter-genepool cross, as the parents of the mapping population are squarely in opposite genepools as shown in the neighbor joining dendogram of Blair et al. [28]. Segregation data and the software program MapDisto v. 1.7 beta with a LOD > 3.0 were used to place the new SSR loci in the previously established genetic map for DOR364 × G19833 [8,10].

Genetic distances were derived from recombination fraction based on the Kosambi function. To integrate the physical and genetic map the BAC contigs found in the *Phaseolus *WebFPC database http://phaseolus.genomics.purdue.edu/ were integrated with the BES-SSR loci on the newly-constructed genetic map.

## Abbreviations

BAC: Bacterial Artificial Chromosome; BES-SSRs: microsatellite markers from BAC-ends; FISH: Fluorescence *In Situ *Hybridization; PCR: Polymerase Chain Reaction; RIL: Recombinant Inbred Lines; SSR: microsatellite locus; STC: Sequence-Tagged-Connector;

## Authors' contributions

JMC participated in planning of the studyn, carried out the SSR genotyping, constructed the integrated map and drafted the manuscript. MCC helped with the microsatellite analysis. JJS designed the SSR primer pairs and SAJ provided funding for SSR design. MWB conceived of and coordinated the study, obtained overall funding for the study and co-wrote the paper. All authors read and approved the manuscript.

## Supplementary Material

Additional file 1 **Information about the BMb microsatellite loci used for primer pair development**. Primer sequences, expected product sizes, SSR motifs and repeat length, contig identification, number of clones for each contig along with the PCR program used for each primer pair. Click here for file

Additional file 2 **Information about the components of the integrated common bean map**. SSR markers, BAC clones and contigs included in the integrated genetic and physical map of common bean with contig size. Click here for file
