# Supplementary material for: Integration of physical and genetic maps of common bean through BAC-derived microsatellite markers
Source: BMC Genomics. 2010 Jul 16;11:436. doi: 10.1186/1471-2164-11-436 (PMC3091635; doi:10.1186/1471-2164-11-436)
Supplement: Additional file 1 — Information about the BMb microsatellite loci used for primer pair development. Primer sequences, expected product sizes, SSR motifs and repeat length, contig identification, number of clones for each contig along with the PCR program used for each primer pair. [file 1471-2164-11-436-S1.DOCX]

| **BMb marker** | **Sequence ID** | **Left Sequence** | **Right Sequence** | **Expected Product Size** | **Motif and repeat lenght** | **Contig ID** | **Number of BAC clones** | **PCR program** |
| --- | --- | --- | --- | --- | --- | --- | --- | --- |
| BMb2 | PV_GBa0032N21.f | AATGTCTTTCCGACACATTC | GACCAATTGATTTAGTTCGG | 270 | (AT)13 | 4 | 161 | TD53-45 |
| BMb10 | PV_GBa0012B21.r | GAGGCAATTCGTTTGAAATA | AGACAATCCGTTATACAATCT | 225 | (AT)14 | 11 | 89 | TD52-45 |
| BMb15 | PV_GBa0086G10.r | TCTCTCTTCATTATATAGCCACA | TCTTTATTGCAACAATCCAA | 267 | (ATA)34 | 13 | 94 | TD51-42* |
| BMb23 | PV_GBa0109E17.r | TACCGACAACTCATCAGTCA | TTCCGCCAGTTATGTTAGTT | 257 | (TA)13 | 19 | 130 | TD52-45 |
| BMb25 | PV_GBa0040B19.r | TCGGGTAGAAATGTTCCTTA | AATTGTAACAGCGTAAACAAA | 198 | (GA)7 | 20 | 67 | TD52-45 |
| BMb31 | PV_GBa0114D05.f | CTGATGGAGCATTTGATGTT | CCTAAGGTTTAACTTGCTCTG | 247 | (TA)24 | 26 | 118 | TD54-47* |
| BMb32 | PV_GBa0078O18.f | CTGACCTCGATCTTTCTGAG | GAACCATCCAGTTAAACCAA | 253 | (AT)22 | 27 | 322 | TD54-46 |
| BMb35 | PV_GBa0078D04.f | CCTTCATTTGAATTGTTGGT | CAATGTTCAATCAAATGTGC | 193 | (TC)14 | 28 | 31 | TD52-45 |
| BMb43 | PV_GBa0023B15.f | GTGATCGGCTACATTAGCAT | GCTCTCATGTTCTCTTTCTCA | 143 | (TA)10 | 34 | 21 | TD55-48 |
| BMb45 | PV_GBa0093F02.f | GATTCACCACGTCAAACTCT | ATTCCACGCTCATACAGAAG | 270 | (TC)12 | 39 | 164 | TD53-42 |
| BMb56 | PV_GBa0015D04.r | CAACGGAGGTATCGGTATAA | CGTCCAAAGGTTAGTCATGT | 288 | (GAA)15 | 45 | 27 | TD55-47* |
| BMb57 | PV_GBa0120J15.f | CAAAGCTTGAACACACTTGA | TTACATAGTGGACGGGATTC | 250 | (TAA)15 | 47 | 23 | TD55-48 |
| BMb64 | PV_GBa0121D23.f | GGT GTT GGC GAT ATA AAG TC | TAT AAT GGA ACC CAT AAC GG | 231 | (TA)21 | 49 | 292 | TD53-45 |
| BMb66 | PV_GBa0092G07.f | AACACAATCCCAATGTTACC | AATAATTCATGATTCCTCGG | 247 | (TC)15 | 50 | 83 | TD51-42 |
| BMb67 | PV_GBa0039F02.r | CCGTAACACTTGGTTAGGAG | GAGATACAGTGGCCTAGCAC | 227 | (TC)13 | 53 | 91 | TD55-48 |
| BMb73 | PV_GBa0112F17.f | AACATGGCTCAAGATAATGG | GTTTACTTGGTGATGGAGGA | 235 | (CTT)7 | 69 | 70 | TD54-46 |
| BMb75 | PV_GBa0105P06.r | AAAGTCTCTTTCTGCGTCAC | TGACCATTATTCATTCCCAT | 280 | (AT)32 | 71 | 187 | TD53-45 |
| BMb80 | PV_GBa0089E11.r | GCCTAATTCGATGCTAAGAA | AAAGAGAGAAAGCAGAGAAA | 152 | (TA)21 | 75 | 90 | TD53-45 |
| BMb81 | PV_GBa0071E23.r | GGTGTTGGCGATATAAAGTC | TAATGGAAGATCCATGTTGA | 271 | (AT)9 | 76 | 310 | TD52-45 |
| BMb83 | PV_GBa0052F24.r | CCAATTGGCGAATACTTTAC | AATACTCATATGCAATAGTCAAA | 238 | (TA)10 | 77 | 19 | TD51-42 |
| BMb84 | PV_GBa0094O13.r | GTCACAGCAAACCCTATCC | CTCCATTGACAAAGAGAGAAA | 121 | (AC)6 | 83 | 77 | TD54-46 |
| BMb88 | PV_GBa0083E19.f | ACAACATGATGCAACAAAGA | TATCTCCAGTCTGGCTTCAT | 283 | (TAA)5 | 84 | 83 | TD54-47* |
| BMb96 | PV_GBa0009F12.f | CATAAAGCACGTCACTTCAA | GCCTTGGACACTACCATTT | 126 | (CA)11 | 90 | 66 | TD55-47 |
| BMb97 | PV_GBa0101P22.f | CCTCTCCCTTATCCTCTATCA | GCTGACCCTGAAATTGTATC | 179 | (TC)7 | 91 | 31 | TD55-47 |
| BMb98 | PV_GBa0054A10.f | ACTTGTTGAGTTGGAACCAC | GGCTTGGTGAATCTATTCTG | 177 | (TA)18 | 92 | 435 | TD54-47* |
| BMb99 | PV_GBa0054F21.r | TCCTCTCTAAATCGTCGTGT | GTTGCGTTGAGTTTCTCTCT | 214 | (TC)29 | 93 | 60 | TD55-48 |
| BMb102 | PV_GBa0021I03.f | GGCTGCTTACAAACCATTAC | TTGTAAGCTGTTCTCTCTCTCTC | 120 | (TA)13 | 97 | 90 | TD55-48* |
| BMb106 | PV_GBa0009P10.f | CTCCAACTTCAATTACAGGG | GGATGTGGACATCACTTTCT | 186 | (AT)17 | 101 | 71 | TD54-47 |
| BMb109 | PV_GBa0007I03.r | AGAAATTTCCTCGGGATAAG | CACATGCAACATTTAAGAGG | 236 | (AT)14 | 106 | 333 | TD53-46* |
| BMb121 | PV_GBa0053B05.f | GCCAAAGAGTTGATAGTTGC | GAACTGTTCGATGCTCATGT | 256 | (TA)14 | 111 | 242 | TD55-48 |
| BMb122 | PV_GBa0002I17.r | AAATCTTTACTCTTCATGCATTT | ACTTGCACGGGATAGATTC | 268 | (AT)17 | 114 | 234 | TD53-45 |
| BMb125 | PV_GBa0027P14.r | AGG CAC CGA TAA ATT GAG TA | AGC AAA GTG TCT GAA TTG CT | 229 | (ATA)28 | 117 | 71 | TD54-46 |
| BMb128 | PV_GBa0033F13.f | AGGCACCGATAAATTGAGTA | CCACTGAATCAGCCACTAAA | 253 | (ATT)8 | 118 | 90 | TD55-47 |
| BMb131 | PV_GBa0003K06.f | AAAGAGTGAACCGTTCCTTA | AGGATCACCCTTCTGGTAAT | 129 | (AAT)6 | 130 | 236 | TD55-48 |
| BMb133 | PV_GBa0095A15.r | ACCTCTGAGGGAGAAGATGT | GATAAATGTGTGAATGCCCT | 238 | (AT)14 | 133 | 182 | TD54-47 |
| BMb140 | PV_GBa0118D13.f | CATGCACACTTACAAGCCTA | ATGAATTGGCTAAGTGGAAA | 267 | (AT)10 | 136 | 185 | TD53-46* |
| BMb143 | PV_GBa0070I19.f | TCACTCACTCACTTTCACTCA | AAGATCGTAGTCGCATGATT | 159 | (AT)10 | 137 | 57 | TD55-48 |
| BMb151 | PV_GBa0020B16.f | ATTGATTTATCGCCTTGAAA | AAATGAAACGGAAGAGGATT | 280 | (AT)17 | 142 | 248 | TD51-42 |
| BMb152 | PV_GBa0102J20.f | ACGCAGAGAAATCTCCAATA | CCTTCCATGATTTGTTGTTT | 208 | (AT)18 | 142 | 248 | TD53-45 |
| BMb157 | PV_GBa0109O17.f | TGACTCAAACCAACTTATGC | CATCTTAGTCCTGCTGCTTC | 221 | (AT)14 | 147 | 158 | TD54-47 |
| BMb160 | PV_GBa0064L02.r | TATGCTGACCTTGGAACTTT | GGTAAACTGCAATTGGTCTC | 269 | (AT)14 | 159 | 174 | TD55-48 |
| BMb162 | PV_GBa0085B13.f | CCAACCAGAATTTGAGAAGA | ATAGCTAGGATGGAGGACCA | 106 | (TA)11 | 162 | 129 | TD54-46 |
| BMb172 | PV_GBa0118M17.f | GTTTGGTTTCCCTCTCTTCT | TGGCATCTCTTTCTCACTTT | 297 | (GA)10 | 170 | 144 | TD55-48 |
| BMb174 | PV_GBa0088F02.f | TTGAAACAAATCAGACCCTC | ATACATAGATGCAAGAGCGA | 100 | (TTA)10 | 172 | 26 | TD55-42 |
| BMb177 | PV_GBa0090P09.r | CTGACATGTGTGGAACTCTG | TGAGTTGGTTTCCATAGCTT | 233 | (CT)7 | 174 | 14 | TD55-48 |
| BMb180 | PV_GBa0101N22.r | TTGCAATGTGATCATTTGTT | AACACAGTCAATGGTGTGAA | 251 | (TTA)23 | 179 | 48 | TD52-45 |
| BMb181 | PV_GBa0107A16.r | GAATCCTCGTGGCATATTAG | ACGTGATGATTAGATTGAATG | 193 | (AT)12 | 180 | 69 | TD52-45 |
| BMb182 | PV_GBa0098C22.f | AAATTCCAACCTCTGTGCTA | TTTGGCTGTGATTCTTCTTT | 227 | (TTA)11 | 185 | 36 | TD54-46 |
| BMb185 | PV_GBa0079D15.r | GACATTGGCTCAGTTTCATT | TCATCATCATCTGTTACATCAA | 182 | (TGA)6 | 187 | 33 | TD53-45 |
| BMb188 | PV_GBa0115O12.f | TTGTGTCGTGTAGAGTGTGC | TCTCCATAGCTCAACCATTT | 276 | (GA)8 | 191 | 92 | TD55-47* |
| BMb190 | PV_GBa0124H14.r | CCATGATGATTGTTGTTTGA | AGTGTTTCGGTCGTCATAAT | 212 | (AT)17 | 194 | 155 | TD52-45* |
| BMb191 | PV_GBa0030E19.f | GCTCTGACTCTGGTGTTGAT | TTCCAAAGACAGTTCAGGTT | 194 | (AT)13 | 196 | 135 | TD55-48 |
| BMb192 | PV_GBa0035O13.f | GGTTTGACACCAGTTCATTT | ATTGGAAATGTGTTTGTCAC | 253 | (TA)11 | 203 | 29 | TD53-45 |
| BMb194 | PV_GBa0002G03.f | TCGGGTAGAAATGTTCCTTA | AAGAGGAGATACGAGCATCA | 282 | (GA)11 | 205 | 19 | TD54-47 |
| BMb198 | PV_GBa0009E23.f | GGTGAAGACGACTTATCACA | TCACCAGTAATTGGGTGTTA | 270 | (TTA)6 | 210 | 42 | TD55-47 |
| BMb201 | PV_GBa0110I18.r | TGCAAAGAATCACCTACACA | CACAAACGATCAACTTGCTA | 221 | (TA)34 | 211 | 12 | TD55-47* |
| BMb202 | PV_GBa0101I18.r | AATTTGCATTAATTTAAGACTGC | TTTCGGGTTTCATACAGAGT | 102 | (AT)10 | 212 | 36 | TD53-42 |
| BMb212 | PV_GBa0020M04.f | AGGAAATCTACCTTCATCAGG | TTCTTGTAGTGATGTTTATTCCA | 289 | (TA)16 | 217 | 16 | TD53-46* |
| BMb213 | PV_GBa0025L04.r | TAGTGGCCAAGAAAGTGTTT | TCTGTAGGTGGGTTAGTCGT | 129 | (AT)18 | 225 | 57 | TD55-48 |
| BMb214 | PV_GBa0050L20.r | TGATTAACTCACATCACACGA | GAAGTATCATGGAGGAACCA | 125 | (AT)10 | 226 | 90 | TD55-48 |
| BMb216 | PV_GBa0088N12.f | TTTCTCTTCATCCCACAAAC | TTTGGAATTTGGGAGATATT | 153 | (TA)20 | 226 | 90 | TD51-42* |
| BMb217 | PV_GBa0071E22.f | TGACCATTTAGGCATAGCTT | CAATCAAACCCTTCTTAACG | 228 | (ATA)13 | 228 | 25 | TD53-45 |
| BMb221 | PV_GBa0019N05.r | TGAAAGACAAGAGGGTTCAT | TTGTAGGCACTATTCCGTTT | 223 | (AT)10 | 241 | 78 | TD55-48 |
| BMb244 | PV_GBa0059K16.f | TGAGTTTAATGCCGAGTTCT | ACATCCTACTTCATTCACGG | 216 | (AAT)8 | 266 | 35 | TD55-48 |
| BMb246 | PV_GBa0037M02.f | TCTAACCATAACCAACCACC | AACATTCCGAATATCAATCTTT | 279 | (TA)9 | 270 | 35 | TD51-42 |
| BMb247 | PV_GBa0117B24.r | ATCCTAGGGAGTCATGAAGG | AGAATTGTAACCACACCGAC | 145 | (AT)13 | 272 | 32 | TD55-48 |
| BMb248 | PV_GBa0117F14.r | TTGGAACATATCCTCGATTT | TGTCCTCTTTCTCTATCTCCC | 110 | (AT)22 | 273 | 57 | TD52-45* |
| BMb250 | PV_GBa0018N22.f | TTGGTGGAGGAGAGATTAGA | TGTTTATCATGTTGGAATCCT | 270 | (TA)19 | 274 | 31 | TD53-46 |
| BMb252 | PV_GBa0017K05.f | TTTCATGTGACGAGTCATT | CTTCCACAATCCAGATGTTT | 282 | (AT)15 | 282 | 67 | TD53-45 |
| BMb256 | PV_GBa0121D16.r | CTCCGCACTTACTTAACCAC | GGCATGGTCTTCTTTAGTTG | 234 | (AGA)25 | 294 | 41 | TD55-48 |
| BMb257 | PV_GBa0005N06.r | GTCAATGAAGGCATCACTCT | ATGTCCTCCATAGCACATTT | 219 | (TA)15 | 305 | 15 | TD55-48 |
| BMb259 | PV_GBa0083O12.f | GGATAGTGAGGACTGCTTTG | TTCATCCCTCATATTATCGG | 272 | (AT)19 | 311 | 47 | TD53-42 |
| BMb262 | PV_GBa0066K18.f | ACTAAGAAGTCGCACACGAT | AGGCACTAATACTGCTTTGC | 261 | (AT)12 | 327 | 21 | TD55-48 |
| BMb264 | PV_GBa0070L10.f | TGTACGTATGTTTGCAATGG | TGATCAAAGGAATCAACAAA | 182 | (AT)13 | 331 | 11 | TD51-42 |
| BMb266 | PV_GBa0112M06.f | AAATTCAAACCAGCCATTC | GGCAATTACATTTGGAGAAA | 156 | (TAA)6 | 342 | 9 | TD52-45 |
| BMb267 | PV_GBa0016G01.f | TGAGCATCCTCTACTTGGTT | AATCTCGCCTCTCTCTCTTT | 224 | (GAA)9 | 346 | 58 | TD55-48 |
| BMb268 | PV_GBa0026M15.r | ATCACCCATTGGTAAACTGT | ACATAAGAGACCAAACTCCAA | 278 | (TA)10 | 349 | 145 | TD55-48* |
| BMb275 | PV_GBa0060H14.r | CTT CAC AAG CTG GGA TAC AT | GGA TTC AAT TCG TCT GTT GT | 210 | (AT)16 | 358 | 239 | TD53-45 |
| BMb276 | PV_GBa0104O23.r | TTT CAT GTG ACG AGT CAT T | AAC ATG GAA GTC TCT CAA CC | 234 | (TA)22 | 358 | 239 | TD51-43 |
| BMb277 | PV_GBa0112C01.f | GTAGGTCAACTCGGCTATTG | GCTTTATGCACACGTTTATTC | 238 | (TA)28 | 359 | 41 | TD54-46 |
| BMb279 | PV_GBa0106M10.f | TCGTGTTAACCTTGCAATAA | AAACGGTTATAAGGAGAATC | 288 | (AT)12 | 367 | 13 | TD51-42 |
| BMb283 | PV_GBa0034I11.r | ATAAAGAATTGCTCTGGGTG | TCGAATGTCAGAGGTACACA | 164 | (TA)8 | 375 | 28 | TD54-46 |
| BMb287 | PV_GBa0069L21.r | CGTGTGACACTCGTATTGAC | GCAACAAACACAGAAACAAC | 233 | (TAT)5 | 381 | 16 | TD55-47* |
| BMb290 | PV_GBa0056I21.r | TGGTTGGATTTGGAATTTAT | AACATTAGGCAAGAAACCAA | 150 | (AG)12 | 384 | 29 | TD51-42 |
| BMb292 | PV_GBa0054A12.f | GTCCTTGAAGACTCTTGTGC | GTTTCGCCAGTTATGTTAGC | 178 | (AT)14 | 385 | 93 | TD55-48* |
| BMb293 | PV_GBa0089L04.f | CAATTCTACACTTTGGTGGG | AACGTCATTGATTTGACTCC | 154 | (CTT)7 | 389 | 197 | TD54-47 |
| BMb296 | PV_GBa0078P23.r | AGCGTGGTATGATCAGATTC | AAGAGGAGATACGAGCATCA | 249 | (GA)10 | 395 | 61 | TD55-48 |
| BMb297 | PV_GBa0122G09.f | TTGGGTAATGAACAGGAAAC | ATGATTAGGAGGCATACAGG | 234 | (TA)11 | 396 | 110 | TD54-46* |
| BMb302 | PV_GBa0075B08.f | TTTGGATGATGTTTGATTATTG | ATTAATCCGTGGGTGTTAGA | 228 | (TA)13 | 416 | 25 | TD51-42 |
| BMb305 | PV_GBa0094H17.r | AATTCTGAGATGTCATTGCC | ACAACATTGTCTTAGGCACC | 296 | (AT)25 | 421 | 21 | TD54-47 |
| BMb309 | PV_GBa0071K09.f | TCATTTATTTGAGGGAGGAA | CCAATAAAGGGTCACACAAT | 260 | (AT)25 | 425 | 22 | TD52-45* |
| BMb310 | PV_GBa0006E18.r | TTTGAATTGATAAGGATAAGTTG | TGTACGCGTGTTTGAGTAAG | 209 | (AAT)7 | 426 | 57 | TD51-42 |
| BMb316 | PV_GBa0101K24.r | CAAAGCTAACTCACTCACCC | CCTTGATCAAATTTGTGGTT | 187 | (GGT)6 | 435 | 21 | TD53-45 |
| BMb318 | PV_GBa0033H05.r | AAGCTTGATCTTTGATGGAA | GCTTAACAATTGAGAGGTTGA | 277 | (AT)19 | 436 | 67 | TD53-46 |
| BMb320 | PV_GBa0033D16.f | TTTCTGCATGACAACACATT | ACTTAATCCGAAATCGTTCC | 225 | (AT)30 | 441 | 55 | TD54-46* |
| BMb329 | PV_GBa0119H11.r | CCGGTGAGATAGTCAACC | GAAATAAAGGATGTTGCCAG | 143 | (AT)18 | 451 | 80 | TD53-45* |
| BMb337 | PV_GBa0020P23.f | TAGAGTTACAGGGATGTGGG | TCAAACATGAACCGTAGTGA | 136 | (AGA)7 | 498 | 24 | TD55-48* |
| BMb339 | PV_GBa0098F02.f | CGCCATAGTTGAAATTTAGG | CGTGAGCATATGTATCATTCTT | 162 | (AT)17 | 502 | 33 | TD53-45 |
| BMb341 | PV_GBa0049L09.r | CATGAAATGCATCGAAATAC | GGGAGATTGATAGGGTGAAT | 226 | (AT)15 | 527 | 17 | TD51-42 |
| BMb343 | PV_GBa0061M20.r | ATGGCTAGTGGAATCACATC | AGTGTGTGTGTGAGAGAGCA | 152 | (AC)10 | 530 | 84 | TD55-48* |
| BMb344 | PV_GBa0093C14.r | AGATTCGGTTCTCTAGGTCC | ATGGTTCTTGGTAGGGAGAT | 122 | (TA)18 | 531 | 134 | TD55-48* |
| BMb349 | PV_GBa0012H20.f | TCAGTAAGAAAGGAGGACGA | TCGTCCATCTTCAAGGTTAG | 269 | (AGA)6 | 566 | 32 | TD55-48 |
| BMb352 | PV_GBa0059C04.r | CGTTGTTGTTCATGCAGATA | ACTGTTCCTTCCTCTTCTCC | 244 | (GA)13 | 570 | 28 | TD55-47 |
| BMb353 | PV_GBa0062L15.f | AAATGCTGTAGAGCCAACAT | TGGATTACACAAGGGTCTTC | 278 | (TA)14 | 605 | 34 | TD55-48 |
| BMb356 | PV_GBa0094N01.r | TCCGAATTTCTTAATTTCACTT | ATCGCGGATTTATATGTGTC | 187 | (TA)14 | 610 | 29 | TD55-42 |
| BMb357 | PV_GBa0056O21.f | ACATTCAATCGGTTATTTCG | TTTCATCACAGGACACAATG | 228 | (AT)18 | 616 | 132 | TD52-45* |
| BMb361 | PV_GBa0047D11.f | GTTCAGGGAACCTACATTCA | ATCTTGTTTCATTCAACGGT | 215 | (TCA)6 | 643 | 39 | TD54-46* |
| BMb362 | PV_GBa0006J20.r | TATTCTGTCGGGCATTCTTA | CCTCGCTAGAACAATCAAAG | 294 | (TA)15 | 665 | 67 | TD55-47 |
| BMb363 | PV_GBa0055B11.f | AAAGGCATATGACATTCCAC | TTTACATTCAGTTGTTCCTTCT | 212 | (ATT)12 | 673 | 12 | TD54-46 |
| BMb364 | PV_GBa0100N18.f | TAGGAAATGATTCCCAATGT | GCTGTGGCATATGTGTTATG | 148 | (TA)14 | 676 | 34 | TD53-45* |
| BMb365 | PV_GBa0064J21.f | GCAGAAGGAACTTTGCTAGA | TTTAAATCTCCCTGCACAT | 261 | (TA)14 | 677 | 11 | TD53-45 |
| BMb366 | PV_GBa0114E20.f | TTTGGACTCATTCAACACAA | CATTTACGGTTATGATCCCT | 211 | (TA)13 | 679 | 44 | TD53-46* |
| BMb369 | PV_GBa0113M16.f | TCTCTTCCGTTAACCAACAT | ACCTTACTTGAGAACCCAAA | 178 | (AT)16 | 686 | 95 | TD55-48 |
| BMb371 | PV_GBa0046E07.f | CAATAGGTGTTTCTCAAGCC | ACAAATGACATGAACAAGCA | 125 | (GA)6 | 691 | 33 | TD54-47 |
| BMb372 | PV_GBa0106L16.r | TTTCATATCTCCATCGGATT | TTTGGATCTTGGGTCTAAAT | 140 | (AT)24 | 702 | 6 | TD52-45* |
| BMb373 | PV_GBa0027N15.r | GAATATGAACCAGCACCTGT | CACTTGTTGCTGTTGATGAC | 183 | (CA)13 | 704 | 72 | TD55-48 |
| BMb379 | PV_GBa0092P08.r | TTTGCAACCTCTACTCATGT | TTGAGGTGTCAACATAGCTG | 274 | (AT)12 | 725 | 24 | TD55-48 |
| BMb381 | PV_GBa0055B15.f | TGGAAATTGTCATTAACTTTGA | CTCAGAGATGGAAGGAGTGA | 113 | (AT)20 | 744 | 65 | TD52-45* |
| BMb386 | PV_GBa0108G03.r | CCAAATAGTAGGGTGAACCA | ATTGGAAGAGGTGCAACTAA | 266 | (AT)20 | 769 | 76 | TD55-48 |
| BMb388 | PV_GBa0042E01.r | TCCAAATAGAACAATTGGAAA | TTTGTTACCCTTTCTAGAATAAA | 275 | (TA)22 | 789 | 30 | TD51-42* |
| BMb389 | PV_GBa0009D07.r | TGCATCCAGAAGTCAACATA | TGTAAACTCCGTATCCATCC | 280 | (TA)9 | 796 | 11 | TD55-48 |
| BMb396 | PV_GBa0098A14.f | ATCATTCGTCTGCCTAGAAA | ATAAAGTTTGCAGTTGGTCG | 251 | (TAT)9 | 821 | 103 | TD55-47* |
| BMb401 | PV_GBa0023H15.f | ATCGCGGTTTCTAGAACATA | TCAACTTTGTTAGGTGGGTT | 265 | (AT)11 | 849 | 7 | TD55-47* |
| BMb405 | PV_GBa0043K15.r | TGATTCTTTCAAATTGAGCA | TGCAACCTAGCTCCTTTAAC | 237 | (AT)19 | 873 | 39 | TD51-42 |
| BMb408 | PV_GBa0007K12.f | ATGGCCGATGACTTATTACA | TTTACCACTAGGAATTGATAGGA | 294 | (TTA)9 | 879 | 10 | TD54-47* |
| BMb414 | PV_GBa0032P15.f | AACGACCATTCGTAGAAAGA | GCTCACAAATACGTCGTAGG | 218 | (CTT)8 | 924 | 44 | TD55-48 |
| BMb415 | PV_GBa0039N04.r | TTTCTAAATGTTGTTTACTCCAA | AAATACCCTACTCAGAGCCC | 174 | (TAA)14 | 970 | 31 | TD52-45 |
| BMb416 | PV_GBa0086P03.f | CTACCGTCCTCTCTTCCTCT | GATGAAAGCTTATGGAGGTG | 281 | (AT)20 | 982 | 41 | TD55-47 |
| BMb419 | PV_GBa0005N16.r | ACAGACGTGACAACAACTTT | TCCATATTTGCATACTTTATGTT | 271 | (TAA)16 | 1001 | 10 | TD53-42 |
| BMb420 | PV_GBa0051I15.r | CAGTTTATACCTGAGCAGCA | AAGGACGAAGACACAAGAAA | 296 | (TA)9 | 1022 | 37 | TD55-48 |
| BMb422 | PV_GBa0099J10.r | ATTCGAATTTGGATCTGATT | ACTTCACAAACGCTGTCTCT | 138 | (TAA)6 | 1032 | 30 | TD51-42 |
| BMb424 | PV_GBa0101K19.f | TTGAGGGAGAAGATGGAAAT | GATAAATGTGTGAATGCCCT | 253 | (TA)20 | 1042 | 16 | TD54-47 |
| BMb427 | PV_GBa0102C11.f | AGAGATGAATCTTATCGCTAACT | AACTCCCTCGAATCTTATCC | 268 | (TAT)9 | 1083 | 14 | TD55-47 |
| BMb428 | PV_GBa0087M09.f | TCG GGT AGA AAT GTT CCT TA | CAT GCA TTC TTT CTT CTT CC | 148 | (GA)10 | 1099 | 39 | TD55-47 |
| BMb431 | PV_GBa0002G20.f | TCACGTTATTGGGAGTCAA | ACAAGTCAATTATTACGTTTGAA | 159 | (TA)19 | 1106 | 18 | TD52-45* |
| BMb432 | PV_GBa0021D19.r | TCGTCAATTACGAACAGTCA | ACCTTTCCTCTTCTCCTCAC | 268 | (AG)15 | 1108 | 32 | TD55-48 |
| BMb443 | PV_GBa0002P23.r | TACTTGTTGGCTTCTCCATT | CCACTTCATTTGGTCTTCAT | 295 | (TTA)7 | 1138 | 11 | TD54-47 |
| BMb445 | PV_GBa0115K20.r | CCAAGCTCTGAATCAATCAT | CCAAGTTAACAAATTGAGCC | 151 | (TA)9 | 1142 | 10 | TD53-46 |
| BMb446 | PV_GBa0072F11.r | CGTCTATCTACTCCAGCACC | TCATCTGTGCAAACCAAATA | 291 | (TC)10 | 1147 | 34 | TD53-46 |
| BMb447 | PV_GBa0060K06.r | ATACCCTCCCTAATCTCAGC | ATGTTATTAGTGTTTGCAATG | 268 | (TA)6 | 1154 | 18 | TD55-42 |
| BMb448 | PV_GBa0030L11.r | TCATTTGAGTTAATGGTCCC | TCAATCACACCAGCTTAATTT | 296 | (AT)13 | 1158 | 30 | TD53-46* |
| BMb449 | PV_GBa0121A13.r | GAGATAGGATGGATCGTGAA | GCGGACAAAGAAGTTGTAGA | 298 | (AAT)6 | 1179 | 78 | TD55-47 |
| BMb461 | PV_GBa0044M01.f | GTTGGCGTTCCATCTGTAT | TAAGGGCAGAATCCAACTTA | 232 | (TA)16 | 1301 | 10 | TD55-47 |
| BMb466 | PV_GBa0008K20.f | TGCTTGACAATTACGAGATTA | TTTACATTCCTTACGTTTGGT | 296 | (TA)16 | 1364 | 22 | TD53-46* |
| BMb467 | PV_GBa0074P15.r | AACCCGTTTCTATTCTTTCC | TGATGATAGGACCAATTTCC | 276 | (AT)16 | 1404 | 30 | TD53-46* |
| BMb468 | PV_GBa0066A10.r | TCGGGTAGAAATGTTCCTTA | GTAGCAGCGTGTTTGTTAAA | 188 | (GA)6 | 1419 | 16 | TD54-47* |
| BMb469 | PV_GBa0015K19.f | CATTCATGTGAACCTTTCATT | ATTGTTTGGTTTGTGCTTCT | 145 | (AGA)10 | 1428 | 6 | TD53-45 |
| BMb470 | PV_GBa0044G06.f | ATTTCCTTTGACAACTTCCA | GAAGCGAAGTAATGCCTAAA | 191 | (TA)25 | 1433 | 37 | TD53-46* |
| BMb472 | PV_GBa0074H14.r | GTTTCATGATCAAGGTGCAT | AGACGCTTTCTCATAATCCA | 299 | (TA)13 | 1440 | 23 | TD55-47 |
| BMb473 | PV_GBa0045A18.f | ACAGTGACGGATCTTAAACAA | GTTTGTATGAGGACATCGGT | 166 | (TA)10 | 1442 | 18 | TD55-48 |
| BMb474 | PV_GBa0042G21.r | TTGCTGGAAAGTAACCCTAA | TCCGTTTAATACATCCAAGG | 132 | (CT)7 | 1443 | 27 | TD53-46 |
| BMb477 | PV_GBa0120J07.f | AAAGCCAACAAAGGACTTCT | AAAGTTTGAAATATACGTTGGG | 262 | (AT)19 | 1447 | 9 | TD52-44 |
| BMb475 | PV_GBa0071O24.f | AGCTAACCTTCATGTGGAAA | CCATGAAGAGAGAACTGGTG | 209 | (CT)10 | 1443 | 27 | TD55-48 |
| BMb483 | PV_GBa0049A09.r | ACATCCACCACAATTGATTT | CACACTTCAGACAACGACAC | 158 | (TA)13 | 1453 | 30 | TD54-46 |
| BMb484 | PV_GBa0093J21.r | CAGCTAAACTTTCCGATTTG | TGACTCAAACCAACTTATGC | 172 | (AT)14 | 1458 | 18 | TD53-46 |
| BMb485 | PV_GBa0095B13.r | TATCCCACAACACTTTCACA | AAAGCCTCACCTAGGAAATC | 180 | (AT)12 | 1478 | 20 | TD55-48 |
| BMb488 | PV_GBa0088O15.f | TTGCTTATTGTTTCCGATTT | AAGCCTTGCAAAGAGTTAAA | 236 | (AT)16 | 1487 | 24 | TD51-42 |
| BMb489 | PV_GBa0060I14.f | ACAAACCTTTATTGACCAGC | AAATCAATATCCTTGACTGAAAG | 158 | (TA)23 | 1497 | 13 | TD53-45 |
| BMb490 | PV_GBa0021E07.f | CTTCACAAGCTGGGATACAT | TCCACAACTTTGATGGGTAT | 167 | (AT)14 | 1514 | 3 | TD55-48* |
| BMb492 | PV_GBa0102E06.f | TTCAGTTTGACAAGAACAGGT | TGCATTCTTTGTACTACATTTCA | 242 | (TA)17 | 1538 | 6 | TD54-47* |
| BMb493 | PV_GBa0021L23.f | TCCATAGACACCTACCCTTG | AACAAGCTCTGCTGACAAAT | 174 | (AGA)8 | 1544 | 19 | TD55-48 |
| BMb495 | PV_GBa0068N03.f | GCTATGTGTGATTGCAGTTG | ACAAGAAAGGAAAGGTGAAA | 264 | (TA)9 | 1579 | 31 | TD53-46 |
| BMb497 | PV_GBa0024O12.f | AACACAAGCAACGCTAATCT | GAGTCACCATCACCTTTGTT | 250 | (AT)14 | 1593 | 13 | TD55-48 |
| BMb500 | PV_GBa0110G13.f | GTTCACACAGAAACTGGGTT | ACGTGTCAATCCATTCTTTC | 278 | (TTA)7 | 1598 | 29 | TD54-47 |
| BMb501 | PV_GBa0022J06.r | CTCTGTACTTCTTCCCAACG | GTAGGACGTTGAAGTTCTCG | 207 | (CGA)5 | 1602 | 13 | TD55-48 |
| BMb502 | PV_GBa0074D08.f | ATTCTCAGGCAGGAAACATA | ACGACCCACAATCACTTAAA | 247 | (AC)25 | 1626 | 14 | TD55-47 |
| BMb504 | PV_GBa0113G07.f | TGGTTGAAGAGGTAGTTCGT | TGATACCTCCTACAATTGGC | 186 | (AGC)6 | 1631 | 16 | TD55-48 |
| BMb505 | PV_GBa0094B14.r | AGAATAAATGGTCCCTTGGT | TCAGGATTCTCACACAATCA | 299 | (AT)15 | 1635 | 22 | TD55-47 |
| BMb506 | PV_GBa0120O24.f | GGTACTTGAGAGGGATCAGA | TCAGTCCACAGAAATCATCA | 234 | (CT)11 | 1642 | 28 | TD55-47 |
| BMb508 | PV_GBa0091B21.f | TTGAGACAAATGACTCACCA | CGTGTTCCTTTAAACAATCC | 250 | (CTC)7 | 1648 | 18 | TD53-46 |
| BMb509 | PV_GBa0118A01.f | GTCGAGATGTCAAGTTTCGT | CACCGATCATAACTTGGATT | 275 | (TA)36 | 1659 | 12 | TD54-46* |
| BMb510 | PV_GBa0045O03.r | GCTATTGGAACTCATAACCG | TTCACTCTCTCTCTCTCTCTCTC | 252 | (AT)19 | 1662 | 9 | TD55-42* |
| BMb511 | PV_GBa0026N08.f | TCTCCCTTTGTCATGGTATC | TATGATAGGCAGTCGAGGTT | 234 | (AAT)7 | 1678 | 29 | TD55-48 |
| BMb513 | PV_GBa0089F16.f | CAAAGGCCTAGGATCTACAA | CCTTCACAACTACTGCTACAAA | 244 | (TA)19 | 1690 | 13 | TD55-48 |
| BMb516 | PV_GBa0124O01.r | AGATCGAATCCCTATTGGTT | AAGGTCCACGAAATACAAGA | 217 | (TAA)18 | 1695 | 14 | TD54-47 |
| BMb519 | PV_GBa0068O14.r | CGGGAACCTAGACACACTAC | TGATTTACCTTAGCTTCCCA | 216 | (GAA)11 | 1747 | 29 | TD55-47 |
| BMb521 | PV_GBa0039J03.r | AACACAGGGCAAATTACTGT | TTGAGTGATACGCAGAAAGA | 223 | (TA)11 | 1782 | 20 | TD55-48 |
| BMb522 | PV_GBa0075B11.f | TTACATTTGCATTGTACCGA | GATTCCGATTTCCTCTCTCT | 194 | (AAG)8 | 1784 | 16 | TD53-46 |
| BMb526 | PV_GBa0121L07.r | AAAGGGCAAGTTAGATGTGA | TTTGAAGAATAGAAATCATACTG | 220 | (TA)15 | 1832 | 9 | TD51-42 |
| BMb527 | PV_GBa0089H04.r | CCTATCCCTTTGACCATACA | GAAATCATGGAGACAGCAAT | 218 | (AT)13 | 1833 | 40 | TD54-47 |
| BMb528 | PV_GBa0065L03.r | CAAATTCATAAGCGTCACAA | TGGAGGTATCGAATAATCACA | 120 | (AT)18 | 1836 | 8 | TD53-45 |
| BMb529 | PV_GBa0059P23.r | GCTGCCAATTATCTAGGATG | ATCTTGGCTCAATACACCAG | 182 | (TC)9 | 1845 | 8 | TD54-47 |
| BMb531 | PV_GBa0023M10.r | CAA ATT CAT AAG CGT CAC AA | CCA AGA ATG ATT TAG TGG GA | 273 | (AT)13 | 1869 | 37 | TD54-46 |
| BMb532 | PV_GBa0057B15.f | GCT GCC AAT TAT CTA GGA TG | CAT ATT TCC TCG TCC ATG AT | 268 | (AT)16 | 1869 | 37 | TD54-46 |
| BMb535 | PV_GBa0021P06.r | TCATCAAGTTGCACAAAGAG | TTCCATGAGACCAAAGAATC | 224 | (AT)22 | 1874 | 8 | TD53-46 |
| BMb536 | PV_GBa0048M24.f | AGCGACACGTGTAGAAAGTT | AATGGTGATCATTTGTTGTTT | 287 | (TA)10 | 1876 | 4 | TD52-45* |
| BMb538 | PV_GBa0114O24.r | TTATAACTCTGGGCCTGGTA | GTGGATCCGTACATAATTAGC | 275 | (AT)12 | 1883 | 8 | TD54-47 |
| BMb539 | PV_GBa0118E11.r | TTCCAGGTTATTTCATTTCTTT | TGAGTTATGAGTTAAGTACAGCA | 175 | (AT)6 | 1891 | 19 | TD55-42 |
| BMb540 | PV_GBa0031L15.f | ATTTGGGACAGAATGTGAAG | AATGAGCTTTGAGTGGCTAA | 288 | (GT)16 | 1898 | 19 | TD54-47 |
| BMb545 | PV_GBa0066H07.f | CGTCAAACAAGATCCATACA | GTCAATCCCTTAAGTCGTGA | 230 | (TA)22 | 1931 | 10 | TD54-46 |
| BMb547 | PV_GBa0123L18.r | CCATGTTGTTGGAGAGAAGT | CCGTTAATATTTCGTTAGTGC | 215 | (AT)24 | 1979 | 11 | TD53-46* |
| BMb548 | PV_GBa0032P16.r | AAGCCTTGCAAAGAGTTAAA | TGGGTTGAGTAGAGTTGCTT | 250 | (AT)16 | 2031 | 15 | TD54-46 |
| BMb549 | PV_GBa0019E19.r | AAATGAGGGAAGAGATTGGT | ATTCTGTTTCCTTCTGTGGA | 163 | (GA)11 | 2048 | 11 | TD55-47 |
| BMb553 | PV_GBa0016C18.r | CACGTAATCACCAGTTAGCA | TGAGATATTGTTGGTCATACCT | 289 | (AT)14 | 2134 | 25 | TD54-47 |
| BMb554 | PV_GBa0108A14.f | TCTGTCATAAATAAACACTACCA | ACGTAACAAGGAAATGATGG | 155 | (AT)10 | 2134 | 25 | TD53-45* |
| BMb557 | PV_GBa0021L11.f | AGGATTACACCTCTCCTCAAC | TCCTTTGTTGTGGTATCCTC | 268 | (AAT)5 | 2144 | 10 | TD55-48* |
| BMb558 | PV_GBa0003P01.r | TTGGAATGAAATTATTTGGA | CTTAACATTTGTGCATGGAA | 295 | (AT)22 | 2145 | 2 | TD51-42 |
| BMb559 | PV_GBa0037H08.r | ACTAATGGCAACGTCAAACT | GAGGTTCTTCATCCTCAACA | 121 | (TA)11 | 2157 | 7 | TD55-48 |
| BMb560 | PV_GBa0024A04.f | AACTCATGAGGTGAGGTTTG | GAGGAGGAGGGAATCTATTG | 265 | (AT)13 | 2170 | 2 | TD55-48 |
| BMb563 | PV_GBa0115K09.r | TTGCTGCTATGAACTGATTC | CCATGAAACTGACTTTGGTT | 173 | (TA)7 | 2204 | 8 | TD54-47 |
| BMb564 | PV_GBa0071N10.f | ATTACCGTCAAACATGATCC | CAAGCTGTGCCAATATGTAA | 269 | (AT)12 | 2206 | 4 | TD54-46* |
| BMb565 | PV_GBa0013I17.r | AGATCCTTAGCCAATCAACA | AAATAAACGGGAGTTTCAAAT | 269 | (ATT)12 | 2207 | 6 | TD52-45 |
| BMb567 | PV_GBa0026N09.r | AGGAAACTGAAACTGAACCA | TTAACTCTGTAGCCTCTGGC | 231 | (GAA)7 | 2232 | 6 | TD55-48 |
| BMb571 | PV_GBa0117E19.f | CTCCCAAATTTGTTGAAGAG | GTAACAATAGCAGTGAGGGC | 228 | (TA)14 | 2295 | 12 | TD53-45 |
| BMb572 | PV_GBa0078G03.r | GCAGTTGCCTAATTTAGAGTG | TCCGATATGATCCGTTATTC | 142 | (ATC)5 | 2315 | 6 | TD53-45 |
| BMb573 | PV_GBa0081E16.r | GGGAAATGATTCGTCAAATA | CACGTGTTTCCAAGTCTTCT | 203 | (AG)14 | 2327 | 4 | TD51-42 |
| BMb578 | PV_GBa0067N05.f | AAGTGGAGGGTGAAACATTA | CGCTAACGTTATTTCTCCAT | 165 | (AG)12 | 2412 | 4 | TD54-46 |
| BMb579 | PV_GBa0049C09.f | TGCATCAATAACAAGCTCAA | TTCCCAAATGATAGGAACAC | 277 | (TA)9 | 2415 | 10 | TD53-46 |
| BMb580 | PV_GBa0021D05.r | CTATCATGCTCTCTAAATTATCA | AGAGAGAAGAGAAATGGATAGGT | 262 | (TA)17 | 2424 | 10 | TD51-42 |
| BMb581 | PV_GBa0055C23.r | GCTTACGTGCACCTTTATTT | CATTTAGGTCAGCTCGATTT | 266 | (TA)13 | 2427 | 2 | TD54-46 |
| BMb583 | PV_GBa0065J01.f | ATTTCAAATTTCCTTCACCC | AAATGTTCTAATCCCTGTTACT | 160 | (TA)15 | 2449 | 7 | TD52-45* |
| BMb587 | PV_GBa0088M04.r | TTGCAAGTCTCAAACAGTTG | TCACACTCCTTTACCACTCC | 161 | (GAA)6 | 2466 | 15 | TD55-48 |
| BMb588 | PV_GBa0059C05.f | ATTATCCAAACATGATCCGT | TCATACTGAAGTCATGCCAA | 233 | (AT)11 | 2467 | 2 | TD53-45 |
| BMb590 | PV_GBa0069J10.f | TAGTTGTTGGTACGCATCTG | TGACAATCAGACAACTTGGA | 216 | (AT)12 | 2469 | 16 | TD55-48 |
| BMb592 | PV_GBa0098F10.r | TAACTAGCGGAAACATAGGC | TTATGGGAAGATGATGCAAT | 117 | (TA)12 | 2495 | 3 | TD53-45* |
| BMb594 | PV_GBa0012N21.f | ATAATTCAACTCCCGTGCTA | GAAGCAAGAAGAGATGGAAA | 207 | (CT)10 | 2534 | 4 | TD54-46 |
| BMb596 | PV_GBa0034D18.f | CTTAATGGATCCAGGTCTGA | AGTTGGGATTTGAATTTGTG | 116 | (GAA)7 | 2541 | 5 | TD53-45 |
| BMb598 | PV_GBa0070K23.f | TCTGTTCCTTCCGATCATAC | GTAGCACCAGCTTATTGGTC | 293 | (GA)12 | 2543 | 3 | TD55-48 |
| BMb600 | PV_GBa0051J24.r | TAATGATGGTGATGGTTGTG | TTCTCAATCTCTCATGTCCC | 135 | (AG)16 | 2563 | 2 | TD54-46 |
| BMb601 | PV_GBa0078K20.r | ATTGATCGTATGCAAGGTTT | ACCTGCCATAAATAATGTGT | 136 | (TA)19 | 2606 | 4 | TD55-42 |
| BMb602 | PV_GBa0091P19.f | TGACTCAGACCACGTGTTAG | AAGTCGCACTTATTTCCCTT | 296 | (AT)13 | 2617 | 4 | TD55-48 |
| BMb605 | PV_GBa0008K21.f | ACTCGTTAGCTTGTCACCAT | CTACTATGTTCCGCCTTGTC | 182 | (AGT)7 | 2636 | 3 | TD55-48 |
| BMb609 | PV_GBa0070J08.f | GTACGACTGTGGGTGAGAAT | TGCGTAGTTATGATTTCTTAGTT | 139 | (AAT)6 | 2730 | 4 | TD53-46* |
| BMb611 | PV_GBa0090M22.f | TTTCAACCAATATGAAAGCA | TGTTTAATCAACTGCTGCAA | 143 | (TAA)5 | 2765 | 7 | TD51-42 |
| BMb614 | PV_GBa0013K02.f | CCTCTTGAACTTTGGCACTA | GAAGGTGGAAGATCAATGAA | 272 | (AT)22 | 2822 | 5 | TD53-46* |
| BMb615 | PV_GBa0118C23.r | ATGTCAAATCCAACTCCAAA | CATTTCACTCTTAAATCGGC | 292 | (AT)14 | 2829 | 2 | TD53-45 |
| BMb617 | PV_GBa0120M15.f | GCAATTACGATTAGAGCTGG | AATCAGTAGCCGAGAATGAA | 294 | (AT)11 | 2865 | 2 | TD55-47 |
| BMb619 | PV_GBa0070A17.r | GATGGACACACTCACAAACA | TGTGTTCTACCACCAACAGA | 298 | (AT)22 | 2913 | 20 | TD55-48 |
| BMb620 | PV_GBa0111F08.f | TTACCCAGACAAAGGGTTTA | TCCTTGATTCTTAGGTTCGT | 103 | (AT)13 | 2914 | 250 | TD54-47* |
| BMb621 | PV_GBa0105L13.r | TGTGCTTAGTGTGTGAGGAG | TGAATGTCCCTTATTGGAAC | 213 | (TA)20 | 2916 | 23 | TD54-46 |

* multi-banding or unexpected allele size
