# Supplementary material for: Integration of physical and genetic maps of common bean through BAC-derived microsatellite markers
Source: BMC Genomics. 2010 Jul 16;11:436. doi: 10.1186/1471-2164-11-436 (PMC3091635; doi:10.1186/1471-2164-11-436)
Supplement: Additional file 2 — Information about the components of the integrated common bean map. SSR markers, BAC clones and contigs included in the integrated genetic and physical map of common bean with contig size. [file 1471-2164-11-436-S2.DOCX]

| **LG** | **Integration number** | **SSR** | **BAC clone ID^1^** | **BAC clone size (Kb)**^2^ | **Contig ID** | **Contig size (Kb)** | **No. BACs per contig** |
| --- | --- | --- | --- | --- | --- | --- | --- |
| b01h | 1 | BMb356 | PV_GBa0094N01 | 107 | 610 | 280 | 29 |
|  | 2 | BMb83 | PV_GBa0052F24 | 116 | 77 | 416 | 19 |
|  | 3 | BMb194 | PV_GBa0002G03 | 124 | 205 | 188 | 19 |
|  | 4 | BMb513 | PV_GBa0089F16 | 114 | 1690 | 176 | 13 |
|  | 5 | BMb290 | PV_GBa0056I21 | 96 | 384 | 199 | 29 |
|  | 6 | BMb213 | PV_GBa0025L04 | 78 | 225 | 546 | 57 |
|  | 7 | BMb256 | PV_GBa0121D16 | 88 | 294 | 328 | 41 |
|  | 8 | BMb64 | PV_GBa0121D23 | 89 | 49 | 2,026 | 292 |
|  | 9 | BMb405 | PV_GBa0043K15 | 126 | 873 | 401 | 39 |
|  | 10 | BMb473 | PV_GBa0045A18 | 85 | 1442 | 277 | 18 |
| **SUBTOTAL** | |  |  | **1,023** |  | **4,837** |  |
| b02d | 1 | BMb469 | PV_GBa0015K19 | 115 | 1428 | 193 | 6 |
|  | 2 | BMb495 | PV_GBa0068N03 | 74 | 1579 | 211 | 31 |
|  | 3 | BMb180 | PV_GBa0101N22 | 115 | 179 | 610 | 48 |
|  | 4 | BMb125 | PV_GBa0027P14 | 114 | 117 | 733 | 71 |
|  | 5 | BMb80 | PV_GBa0089E11 | 118 | 75 | 732 | 90 |
|  | 6 | BMb97 | PV_GBa0101P22 | 124 | 91 | 611 | 31 |
|  | 7 | BMb527 | PV_GBa0089H04 | 96 | 1833 | 438 | 40 |
|  | 8 | BMb420 | PV_GBa0051I15 | 120 | 1022 | 395 | 37 |
|  | 9 | BMb497 | PV_GBa0024O12 | 85 | 1593 | 226 | 13 |
|  | 10 | BMb259 | PV_GBa0083O12 | 103 | 311 | 320 | 47 |
|  | 11 | BMb252 | PV_GBa0017K05 | 84 | 282 | 579 | 67 |
|  | 12 | BMb122 | PV_GBa0002I17 | 97 | 114 | 1,766 | 234 |
| **SUBTOTAL** | |  |  | **1,245** |  | **6,814** |  |
| b03c | 1 | BMb590 | PV_GBa0069J10 | 100 | 2469 | 300 | 16 |
|  | 2 | BMb477 | PV_GBa0063G06 | 86 | 1447 | 232 | 9 |
|  | 3 | BMb247 | PV_GBa0117B24 | 88 | 272 | 311 | 32 |
|  | 4 | BMb508 | PV_GBa0091B21 | 102 | 1648 | 194 | 18 |
|  | 5 | BMb506 | PV_GBa0120O24 | 113 | 1642 | 267 | 28 |
|  | 6 | BMb57 | PV_GBa0120J15 | 88 | 47 | 284 | 23 |
|  | 7 | BMb339 | PV_GBa0098F02 | 102 | 502 | 294 | 33 |
|  | 8 | BMb521 | PV_GBa0039J03 | 120 | 1782 | 265 | 20 |
|  | 9 | BMb2 | PV_GBa0032N21 | 93 | 4 | 1,664 | 161 |
|  | 10 | BMb581 | PV_GBa0055C23 | 65 | 2427 | 118 | 2 |
| **SUBTOTAL** | |  |  | **957** |  | **3,929** |  |
| b04b | 1 | BMb133 | PV_GBa0095A15 | 103 | 133 | 1,415 | 182 |
|  | 2 | BMb66 | PV_GBa0092G07 | 139 | 50 | 580 | 83 |
|  | 3 | BMb353 | PV_GBa0062L15 | 127 | 605 | 425 | 34 |
|  | 4 | BMb571 | PV_GBa0117E19 | 74 | 2295 | 146 | 12 |
|  | 5 | BMb43 | PV_GBa0023B15 | 94 | 34 | 268 | 21 |
|  | 6 | BMb548 | PV_GBa0032P16 | 96 | 2031 | 211 | 15 |
|  | 7 | BMb488 | PV_GBa0088O15 | 87 | 1487 | 225 | 24 |
| **SUBTOTAL** | |  |  | **720** |  | **3,27** |  |
| b05e | 1 | BMb293 | PV_GBa0089L04 | 134 | 389 | 1,45 | 197 |
|  | 2 | BMb349 | PV_GBa0012H20 | 110 | 566 | 294 | 32 |
|  | 3 | BMb121 | PV_GBa0053B05 | 125 | 111 | 1,616 | 242 |
|  | 4 | BMb318 | PV_GBa0033H05 | 79 | 436 | 475 | 67 |
|  | 5 | BMb611 | PV_GBa0090M22 | 105 | 2765 | 117 | 7 |
|  | 6 | BMb560 | PV_GBa0024A04 | 73 | 2170 | 103 | 2 |
|  | 7 | BMb250 | PV_GBa0018N22 | 86 | 274 | 230 | 31 |
| **SUBTOTAL** | |  |  | **712** |  | **4,285** |  |
| b06g | 1 | BMb341 | PV_GBa0049L09 | 90 | 527 | 228 | 17 |
|  | 2 | BMb182 | PV_GBa0098C22 | 103 | 185 | 423 | 36 |
|  | 3 | BMb519 | PV_GBa0068O14 | 93 | 1747 | 259 | 29 |
|  | 4 | BMb539 | PV_GBa0118E11 | 94 | 1891 | 198 | 19 |
|  | 5 | BMb419 | PV_GBa0005N16 | 90 | 1001 | 151 | 10 |
| **SUBTOTAL** | |  |  | **470** |  | **1,259** |  |
| b07a | 1 | BMb489 | PV_GBa0060I14 | 64 | 1497 | 178 | 13 |
|  | 2 | BMb191 | PV_GBa0030E19 | 85 | 196 | 1,121 | 135 |
|  | 3 | BMb526 | PV_GBa0121L07 | 110 | 1832 | 197 | 9 |
|  | 4 | BMb502 | PV_GBa0074D08 | 103 | 1626 | 284 | 14 |
|  | 5 | BMb621 | PV_GBa0105L13 | 149 | 2916 | 294 | 23 |
|  | 6 | BMb428 | PV_GBa0087M09 | 113 | 1099 | 461 | 39 |
|  | 7 | BMb160 | PV_GBa0064L02 | 92 | 159 | 1,464 | 174 |
|  | 8 | BMb601 | PV_GBa0078K20 | 74 | 2606 | 97 | 4 |
|  | 9 | BMb202 | PV_GBa0101I18 | 116 | 212 | 346 | 36 |
| **SUBTOTAL** | |  |  | **906** |  | **4,442** |  |
| b08f | 1 | BMb531 | PV_GBa0023M10 | 92 | 1869 | 393 | 37 |
|  | 2 | BMb445 | PV_GBa0115K20 | 77 | 1142 | 117 | 10 |
|  | 3 | BMb386 | PV_GBa0108G03 | 104 | 769 | 739 | 76 |
|  | 4 | BMb174 | PV_GBa0088F02 | 99 | 172 | 335 | 26 |
|  | 5 | BMb267 | PV_GBa0016G01 | 97 | 346 | 462 | 58 |
|  | 6 | BMb277 | PV_GBa0112C01 | 74 | 359 | 296 | 41 |
|  | 7 | BMb578 | PV_GBa0067N05 | 111 | 2412 | 144 | 4 |
|  | 8 | BMb362 | PV_GBa0006J20 | 149 | 665 | 532 | 67 |
|  | 9 | BMb529 | PV_GBa0059P23 | 90 | 1845 | 230 | 8 |
|  | 10 | BMb559 | PV_GBa0037H08 | 80 | 2157 | 113 | 7 |
|  | 11 | BMb474 | PV_GBa0042G21 | 128 | 1443 | 198 | 27 |
|  | 12 | BMb475 | PV_GBa0071O24 | 84 | 1443 | 198 | 27 |
|  | 13 | BMb266 | PV_GBa0112M06 | 82 | 342 | 158 | 9 |
|  | 14 | BMb553 | PV_GBa0016C18 | 93 | 2134 | 263 | 25 |
| **SUBTOTAL** | |  |  | **1,36** |  | **4,178** |  |
| b09k | 1 | BMb563 | PV_GBa0115K09 | 88 | 2204 | 172 | 8 |
|  | 2 | BMb493 | PV_GBa0021L23 | 122 | 1544 | 161 | 19 |
|  | 3 | BMb143 | PV_GBa0070I19 | 102 | 137 | 537 | 57 |
|  | 4 | BMb598 | PV_GBa0070K23 | 90 | 2543 | 118 | 3 |
|  | 5 | BMb264 | PV_GBa0070L10 | 107 | 331 | 210 | 11 |
|  | 6 | BMb594 | PV_GBa0012N21 | 88 | 2534 | 147 | 4 |
|  | 7 | BMb279 | PV_GBa0106M10 | 96 | 367 | 200 | 13 |
|  | 8 | BMb461 | PV_GBa0044M01 | 93 | 1301 | 163 | 10 |
| **SUBTOTAL** | |  |  | **786** |  | **1,708** |  |
| b10i | 1 | BMb447 | PV_GBa0060K06 | 102 | 1154 | 183 | 18 |
|  | 2 | BMb276 | PV_GBa0104O23 | 120 | 358 | 1,672 | 239 |
|  | 3 | BMb532 | PV_GBa0057B15 | 78 | 1869 | 393 | 37 |
|  | 4 | BMb221 | PV_GBa0019N05 | 80 | 241 | 862 | 78 |
|  | 5 | BMb106 | PV_GBa0009P10 | 82 | 101 | 685 | 71 |
|  | 6 | BMb262 | PV_GBa0066K18 | 100 | 327 | 398 | 21 |
|  | 7 | BMb152 | PV_GBa0102J20 | 122 | 142 | 1,983 | 248 |
|  | 8 | BMb414 | PV_GBa0032P15 | 107 | 924 | 379 | 44 |
|  | 9 | BMb96 | PV_GBa0009F12 | 139 | 90 | 1,17 | 66 |
|  | 10 | BMb302 | PV_GBa0075B08 | 103 | 416 | 228 | 25 |
| **SUBTOTAL** | |  |  | **1,033** |  | **7,953** |  |
| b11j | 1 | BMb32 | PV_GBa0078O18 | 109 | 27 | 2,589 | 322 |
|  | 2 | BMb310 | PV_GBa0006E18 | 93 | 426 | 469 | 57 |
|  | 3 | BMb484 | PV_GBa0093J21 | 117 | 1458 | 259 | 18 |
|  | 4 | BMb185 | PV_GBa0079D15 | 94 | 187 | 441 | 33 |
|  | 5 | BMb10 | PV_GBa0012B21 | 85 | 11 | 670 | 89 |
|  | 6 | BMb588 | PV_GBa0059C05 | 102 | 2467 | 127 | 2 |
|  | 7 | BMb619 | PV_GBa0070A17 | 58 | 2913 | 280 | 20 |
| **SUBTOTAL** | |  |  | **658** |  | **4,835** |  |
| **TOTAL** |  |  |  | **9,87** |  | **47,51** |  |

^1^ According to NCBI; ^2^ Information about BAC clones size, contig ID and contig size is according to WebFPC:phaseolus
